# Morphometric Analysis of the Corpus Callosum According to Age and Sex in Middle Eastern Arabs: Racial Comparisons and Clinical Correlations to Autism Spectrum Disorder

**DOI:** 10.3389/fnsys.2020.00030

**Published:** 2020-06-23

**Authors:** Mohammed Z. Allouh, Mohammed M. Al Barbarawi, Heba A. Ali, Ayman G. Mustafa, Safwan O. Alomari

**Affiliations:** ^1^Department of Anatomy, College of Medicine and Health Sciences, United Arab Emirates University, Al Ain, United Arab Emirates; ^2^Department of Anatomy, Faculty of Medicine, Jordan University of Science and Technology, Irbid, Jordan; ^3^Division of Neurosurgery, Department of Neurosciences, Faculty of Medicine, Jordan University of Science and Technology, Irbid, Jordan; ^4^Basic Medical Science Department, College of Medicine, QU Health, Qatar University, Doha, Qatar; ^5^Division of Neurosurgery, Department of Surgery, American University of Beirut Medical Center, Beirut, Lebanon

**Keywords:** autism, corpus callosum, ethnicity, forebrain, magnetic resonance imaging, Middle Eastern Arab, morphometric measurements

## Abstract

This study sought to examine the influence of age and sex on morphometric measurements of the corpus callosum (CC) within Middle Eastern Arab population, in order to obtain reference data and conduct racial comparisons with previously reported measurements from other ethnicities. Furthermore, it aimed to investigate CC variations that may occur in children with autism. To this end, magnetic resonance images of normal brains were acquired from three different age groups, consisting of children, younger adults, and older adults. Brain images were also acquired from boys with autism spectrum disorder (ASD). The CC length, area, and thickness were measured. The CC length was smaller in children than in the other age groups, but no difference in CC length was found between younger and older adults. The CC area and thickness were greater in younger adults than in children and older adults, and greater in older adults than in children. With regard to sexual dimorphism, the CC area and forebrain volume were larger in male children than in female children. No sex-related differences in CC area or thickness were found in adults. However, the ratio of CC area to the forebrain volume was greater in adult females than in males, owing to the smaller forebrain volume in females. The absolute length of the CC was greater in older adult males than in their female counterparts. In addition, significant differences in CC measurements were found in comparison to measurements obtained from other ethnicities. Lastly, significant reductions in CC area and thickness were found in boys with ASD compared to their neurotypical peers. In conclusion, age and sex significantly influence morphometric measurements of CC in Middle Eastern Arab population. This study points to the presence of racial differences in CC size. Finally, it reveals that children with ASD display a distinct reduction in CC size compared to neurotypical children of the same ethnicity.

## Introduction

The corpus callosum (CC) is the largest bundle of commissural fibers in the human brain. It consists of at least 200–300 million fibers that connect the right and left cerebral hemispheres together (Huang et al., 2005; Sakai et al., 2017). This white matter structure plays important roles in transferring sensory, motor, and cognitive information between the right and left cerebral hemispheres (LaMantia and Rakic, 1990). Most of these fibers provide homotopic connections between mirror-imaged areas in the cerebral hemispheres (Huang et al., 2005). However, heterotopic fibers that connect anatomically and functionally different regions of the cerebral cortex in an asymmetric manner are also present (Mooshagian, 2008).

Anatomically, the CC is divided into four distinct regions, consisting of the rostrum, genu, body, and splenium (Sakai et al., 2017). The genu is the most anterior region, near the frontal lobe, while the splenium is the most posterior area, near the occipital lobe. The rostrum is the inferior backward extension from the genu, and the body is the largest area of the CC, located between the genu and splenium (Jones, 1985).

Several studies had previously investigated whether there are any variations in the CC that could be attributed to age or sex. Weis et al. (1993) reported significant age-related changes in the anterior parts of the CC (genu and anterior segment of the body) and concluded that the total area of the CC decreased significantly with age. Furthermore, several studies have suggested that inter-hemispheric communication is better in the female brain than in the male brain, which has prompted investigations of possible sexual dimorphism of the CC (Holloway et al., 1993; Shaywitz et al., 1995; Giedd et al., 1997, 1999; Dubb et al., 2003; Ardekani et al., 2013). For example, Shaywitz et al. (1995) found that the language functions are more likely to be lateralized in males while they are represented in both cerebral hemispheres in females. Additionally, Ardekani et al. (2013) reported that for pairs of male and female individuals with equal brain sizes and similar ages, the CC mid-sagittal area is larger in the female. However, it remains unclear whether these sex differences are similar across different ethnicities.

It has been reported that the size and shape of the brain differ across races (Zilles et al., 2001). Furthermore, studies conducted in different populations have noted that the morphology of the CC seems to exhibit population-related variations that may be influenced by environmental, racial, and genetic factors (Suganthy et al., 2003; Woldehawariat et al., 2014). For example, differences in CC measurements were observed among Indian, Turkish and Japanese populations (Karakaş et al., 2011). Until now, there has been no information on CC morphometric measurements in the Arab ethnic group.

Furthermore, autism is a developmental neurobehavioral condition characterized by difficulties in social communication and interaction, along with restricted repetitive behaviors (Lai et al., 2014). It is 4–5 times more common in males than females. Autism has been classified into three different levels based on the severity of impairment, with level 1 being the mildest level, requiring minimum support, and level 3 being the most severe level, requiring substantial support (American Psychiatric Association, 2013).

The CC has been the most studied structure in patients with autism spectrum disorder (ASD) since it is speculated that poor brain connectivity in autism may be reflected anatomically in this white matter structure which connects the two cerebral hemispheres (Keary et al., 2009; Lefebvre et al., 2015). It has been hypothesized that the size of the CC is reduced in patients with ASD. However, published findings remain inconclusive with regard to a potential correlation between CC size and autism (Kucharsky Hiess et al., 2015; Lefebvre et al., 2015). Therefore, further research is warranted in order to shed light on this hypothesis, especially in the presence of a wide human neuroanatomical diversity.

To the best of our knowledge, no morphometric analysis of the CC has been performed in any Arabic community. This study aims to assess age- and sex-related morphometric measurements of the CC in Middle Eastern Arabs. This will provide normative anatomical reference data to assist in future diagnostic and disease-related investigations in Middle Eastern individuals. Second, it aims to determine whether there are any significant variations between these measurements and previously reported measurements from different ethnicities. Lastly, this study aims to investigate whether there are any significant differences between normal CC measurements and measurements obtained from patients with ASD.

## Materials and Methods

### Image Selection

This study was performed using images for Middle Eastern Arabs stored in the magnetic resonance (MR) imaging unit at King Abdullah University Hospital (KAUH) in Jordan. The Arabic ethnicity was confirmed by the last family name of the subject images, as most Middle Eastern Arabs use their arabic tribe name as their last family name. The study was approved by the Institutional Research Board Committees at Jordan University of Science & Technology and KAUH (IRB # GM7601). The work described has been carried out in accordance with the code of ethics of the World Medical Association (1964 Helsinki declaration and its later amendments). For this type of study, formal consent is waived.

The MR images were reviewed and analyzed by specialized radiologists and neurosurgeons to identify those with normal findings (e.g., no focal or enhanced brain lesions, a normal ventricular system, no mass or shift in the midline structures, no hemorrhage, and no fluid collection). In total, MR images were collected from 227 subjects and divided into three age groups as follows: children aged 2–10 years (*n* = 57; 29 males, 28 females), younger adults aged 20–45 years (*n* = 100; 40 males, 60 females), and older adults aged 55–80 years (*n* = 70; 34 males, 36 females).

### MR Imaging Protocol

All subjects were scanned using the same 3-Tesla scanning device (Philips, Andover, MA, United States). Sagittal three-dimensional T1-weighted images were obtained for all subjects using the following scanning parameters: 160 sections; slice thickness, 1 mm; echo time, 3.73 ms; repetition time, 8.16 ms; flip angle, 8°; acquisition matrix, 240/240; field of view, 59.3 × 23.4 cm; one excitation; and total scan time, 15 min. Some of the children were sedated for the scanning.

### Identification of Midsagittal Sections

**FIGURE 1:**
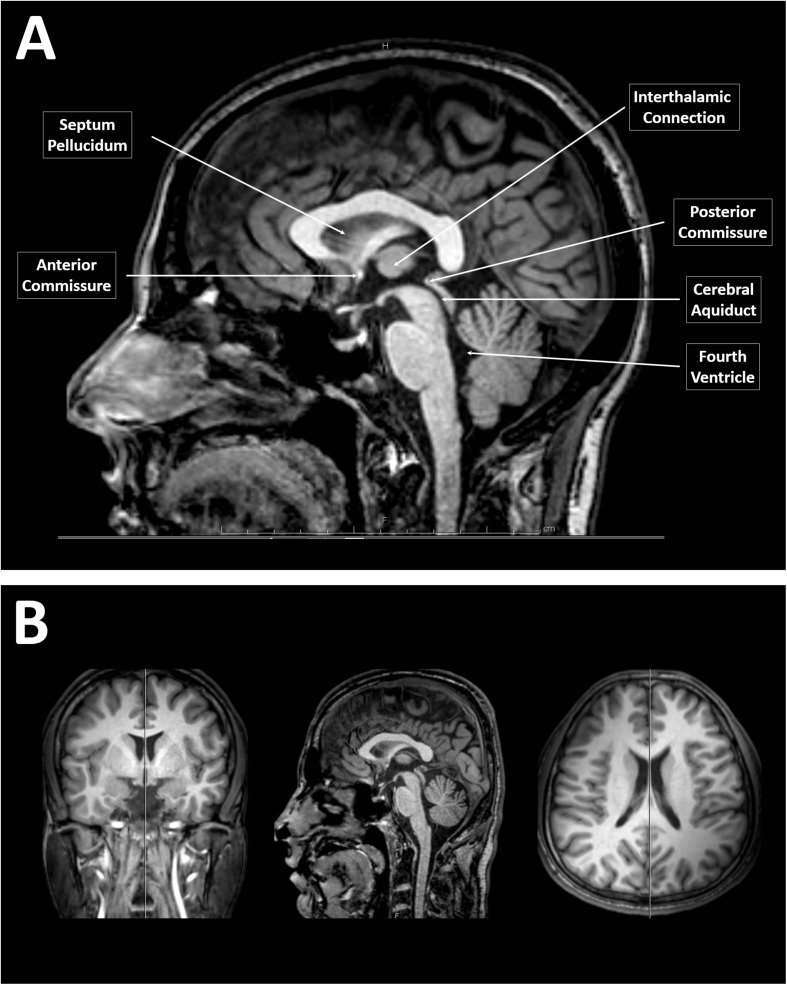
Identification of a midsagittal section from magnetic resonance (MR) images of the brain. **(A)** A midsagittal MR image of the brain showing the clear separation between the tectum and tegmentum of the midbrain by the cerebral aqueduct, a visible fourth ventricle and cerebellar vermis, clear distinction of the septum pellucidum and interthalamic connection, and visible anterior to posterior commissures. **(B)** T1-weighted MR images of the brain of a younger adult male that simultaneously showing the coronal, sagittal, and horizontal series of the sections provided by the Picture Archiving and Communication System software.

All sagittal images were retrieved using the Picture Archiving and Communication System (PACS) viewer. The image closest to the midsagittal section of the CC was selected according to the following criteria described by Pettey and Gee (2002): (i) only traces of cortex visible, (ii) clear separation between the tectum and tegmentum of the midbrain by the cerebral aqueduct, and (iii) good visibility of the fourth ventricle and cerebellar vermis. We also employed the following additional criteria for selecting the midsagittal section: (iv) clear distinction of the septum pellucidum and interthalamic connection and (v) visibility of the anterior and posterior commissures (Figure 1A). Furthermore, viewing the coronal and horizontal series of the sections while simultaneously viewing the sagittal section provided in the PACS software was helpful for determining the true midsagittal section (Figure 1B). All tracing measurements for CC were then performed by two specialized doctors who are very well-trained on using the PACS technology and execute similar measurements continuously on their daily routine work. The inter-rater reliability was evaluated for measurements conducted by both raters, and the average value was more than 0.95.

### Anteroposterior Length Measurements

The anteroposterior length of the CC was measured by tracing a line connecting the most anterior point with the most posterior point of the CC. The length of the CC relative to the total length of the forebrain (length ratio) was also calculated. To calculate the length ratio of the CC, the anteroposterior length of the forebrain was also measured as the distance between the most anterior point in the frontal lobe and the most posterior point in the occipital lobe.

### Area Measurements

The area of the CC was measured using the PACS software. Each area of the CC was traced manually using the region of interest function within PACS. Two manual tracings were made for each case, and the mean value between the two was calculated. All measurements were obtained in a manner which was blind to the sex and age of the subjects.

### Ratio of CC Area to Forebrain Volume

The ratio of the CC area to the forebrain volume was also calculated. To measure the forebrain volume, a stereological method was used to extract quantitative information about the three-dimensional volume of the forebrain from measurements made on the two-dimensional sagittal-sectional areas of the MR images. In this study, the forebrain was defined as all gray and white matter, excluding the cerebellum, brain stem, and all structures below the diencephalon (Bermudez and Zatorre, 2001). Moreover, this study used Cavalier’s principle, which is the most convenient, efficient, and unbiased method of obtaining volume estimations from serial sections (Mayhew and Olsen, 1991). This method includes the following measurement principles: (i) not every section needs to be analyzed – only every fifth or tenth section does; (ii) sections should be equally spaced; (iii) sections that are analyzed should be systematically sampled; and (iv) the first section being analyzed may not be the first section in which the region of interest appears.

There were 160 sagittal MR image sections for each brain. Sections lacking forebrain tissue, primarily the first and last 10 sections, were excluded. The remaining sections were divided into 14 sets, each of which consisted of 10 sections. Here, we uniformly chose to analyze every fourth section from each set. This uniform selection was important for systematic sampling. Each section in the study was 1-mm thick; therefore, each set was 10 mm in thickness. The forebrain area of the chosen sections, including the gray and white matter and the ventricles, was then measured using the region of interest tool in PACS. The cerebellum and brain stem were excluded using a cut line from the level of the superior colliculus to the mammillary bodies. The volume was then estimated by multiplying the sum of the areas by the distance between the selected sections (10 mm) according to the following formula: *V* = *d* Σ *a*, where *V* is the forebrain volume, *d* is the set thickness (distance between selected sections), and *a* is the cross-sectional area.

### Thickness Measurements

**FIGURE 2:**
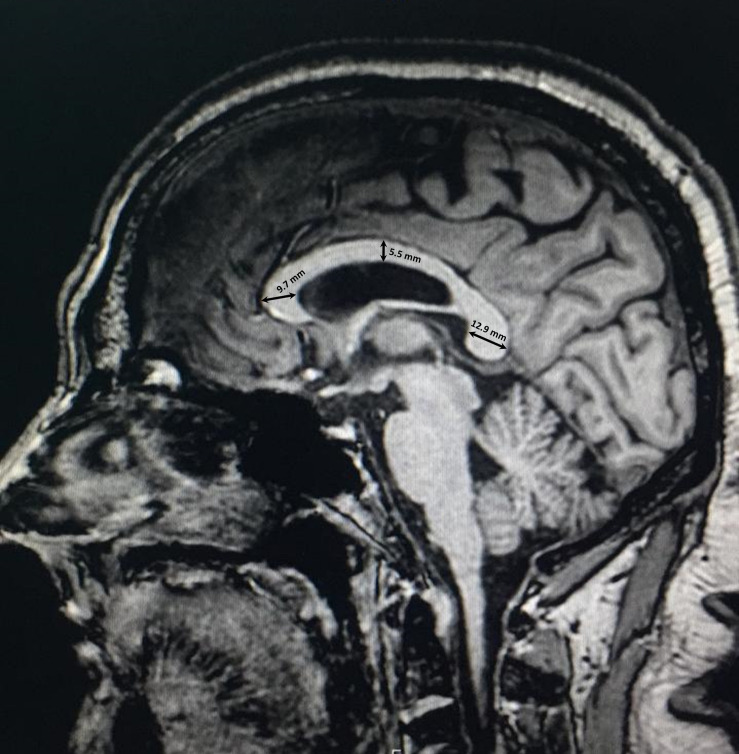
Thickness measurements of the corpus callosum in three regions of the genu, body, and splenium.

The thickness of the CC was measured at three different sites according to the protocol established by Suganthy et al. (2003). First, the thickness of the genu was measured as the distance from the most anterior point of the genu to the most anterior point of the inner concavity of the CC. Second, the thickness of the body was measured as the dorsoventral height at the midpoint of the CC. Finally, the thickness of the splenium was measured as the distance between two points at the dorsal and ventral margins of the splenium at its widest part (Figure 2).

### Racial Comparisons

In order to assess racial variations in CC size, we conducted racial comparisons between our measurements on Middle Eastern Arabs and previously PubMed-reported measurements on other ethnicities. In order to assure unbiased comparisons and to avoid confounding effects, the following inclusion criteria were applied on the selected studies: (i) the study must be performed on midsagittal MR images (studies conducted on brain specimens or used images from different radiological techniques were excluded); (ii) the study must apply similar definitions for the measurements obtained; (iii) the study subjects must be younger adults (aged between 20 and 50 years), as the comparisons were conducted using the younger adult group only; (iv) the comparison must be sex-separated. Based on the aforementioned criteria we were able to retrieve two studies for two different ethnicities from the PubMed database. These studies included Polish (Guz et al., 2019) and Turkish (Karakaş et al., 2011) populations. The compared measurements included the length, thickness (width), and area of CC. Additionally, the anteroposterior length of the forebrain was included in the comparison in order to investigate the variation in the relative length (length ratio) of the CC to the forebrain.

### Correlation With Autism

Mid-sagittal brain MR images were retrieved from KAUH records for 22 male children who were diagnosed with level 2 or 3 autism spectrum disorder. The diagnosis was performed by a clinical expert according to the 5th edition of the Diagnostic and Statistical Manual of mental disorders (DSM-5) published by the American Psychiatric Association, 2013 (American Psychiatric Association, 2013). In summary, the patients had marked to severe deficits in social communication and interaction that included a failure to initiate or respond to social communication, a lack of facial expressions, an inability to understand or use gestures, an inability to make friends and a lack of interest in peers. In addition, the patients displayed repetitive stereotyped motors movements, along with difficulty coping with change and distress when changing their focus or activity.

The boys with ASD were between 5 and 10 years of age at the time of imaging. The CC length, total area, and thickness in the genu, body, and splenium were measured. The findings were then compared with the measurements from the group of neurotypical male children to avoid confounding effects of age and sex.

### Statistical Analyses

All data are presented as the mean ± standard deviation (SD). For the age analysis, the data were organized into three groups according to whether the images were from children, younger adults, or older adults. First, Levene’s test was performed to determine the homogeneity of variance among the three groups. The data were then evaluated with one-way analyses of variance. If a significant difference was found, Fisher’s least significant difference *post-hoc* test was performed to determine the exact statistical differences between the groups. For the sex analysis, the data were divided into two groups according to whether the images were of male or female subjects. Levene’s test for equality of variance was performed first, and the data were then evaluated using independent-samples *t*-tests. The one-sample *t*-test was used to perform the racial comparisons. The measurements from either male or female younger adults were used as the test variables, while their corresponding reported mean values from different ethnicities were used as the test values. Lastly, independent-sample *t*-test was used to determine the statistical differences between neurotypical boys and boys with ASD as described above. All statistical tests were performed at 5 and 1% significance levels.

## Results

### Age-Related Measurements

#### Anteroposterior (Length) Measurements

Significantly smaller anteroposterior lengths of the CC (*P* = 3.3 10^–26^), forebrain (*P* = 0.001), and the ratio of the CC length to the forebrain length (*P* = 5.3 10^–21^) were found in children than in younger and older adults (Table 1). No significant differences in the anteroposterior lengths of the CC (*P* = 0.087) and forebrain (*P* = 0.792) were identified between younger and older adults.

**TABLE 1:** Morphometric measurements of the corpus callosum (CC) in three different age groups of Middle Eastern Arabs.

Measurement	Children (*n* = 56)	Younger adults (*n* = 100)	Older adults (*n* = 70)
**Length measurements**			
Length of CC (mm)	60.55.3**	68.44.0	69.54.1
Forebrain length (mm)	151.510.8**	156.67.6	156.27.5
Length Ratio	0.390.03**	0.440.03	0.450.03
**Area measurements**			
CC total area (mm^2^)	427.883.1^a^	583.483.8^b^	493.477.8^c^
Forebrain volume (cm^3^)	1086.2126.7^a,b^	1123.8118.4^b^	1061.0109.5^a^
Ratio (CC area/forebrain volume)	0.390.07^a^	0.520.07^b^	0.470.07^c^
**Thickness measurements**			
Genu thickness (mm)	8.81.7	10.91.4**	9.21.8
Body thickness (mm)	5.01.0	6.20.8**	5.30.8
Splenium thickness (mm)	13.52.4^a^**	16.62.3^b^	15.62.1^c^

Values are represented as the mean ± standard deviation. ** P < 0.01 significantly different from other groups. Different letters (^a, b, c^) indicate significantly (P < 0.05) different groups (ANOVA, LSD post-hoc).

#### Area Measurements

Significant (*P* = 3.1 × 10^–24^) differences in the total area of the CC were identified among the three age groups (Table 1). The CC area was significantly smaller in children than in younger (*P* = 3.4 × 10^–24^) and older (*P* = 1.1 × 10^–5^) adults and was significantly (*P* = 2.1 × 10^–11^) greater in younger adults than in older adults. A similar trend was found with regard to the ratio of the CC area relative to the forebrain volume (Table 1). The forebrain volume was significantly greater in younger adults than in older adults (*P* = 7.4 × 10^–4^). However, no significant (*P* = 0.231) difference in forebrain volume was observed between children and older adults (Table 1).

#### Thickness Measurements

The thickness of the CC in the genu was significantly greater in younger adults than in children (*P* = 3.7 × 10^–13^) and older adults (*P* = 4.0 × 10^–10^), while no significant (*P* = 0.143) differences were found between children and older adults. The thickness of the CC in the body was significantly greater in younger adults than in children (*P* = 4.3 × 10^–13^) and older adults (*P* = 1.9 × 10^–10^), while no significant (*P* = 0.183) differences were found between children and older adults. In the splenium, the CC thickness was significantly larger in younger adults than in children (*P* = 3.4 × 10^–14^) and older adults (*P* = 0.009), and significantly (*P* = 3.7 × 10^–7^) greater in older adults than in children (Table 1).

### Sex-Related Measurements

#### Measurements in Children

There was no significant (*P* = 0.485) difference in the age between male and female children. The mean ages for male and female children were 5.4 ± 2.1 and 5.0 ± 2.4 years, respectively. No significant differences in the absolute (*P* = 0.052) or relative (*P* = 0.533) lengths of the CC were found between male and female children. The mean length of the CC was 61.8 ± 4.4 mm in males and 59.1 ± 5.9 mm in females. However, the forebrain length was significantly (*P* = 0.003) greater in male children than in female children. The mean forebrain lengths in both male and female children were 155.6 ± 9.7 mm and 147.2 ± 10.3 mm, respectively.

The total area of the CC was significantly (*P* = 0.003) greater in male than in female children. The mean area of the CC was 459.5 ± 85.4 mm^2^ in males and 395.0 ± 67.7 mm^2^ in females. In addition, the forebrain volume was significantly (*P* = 3.0 × 10^–7^) greater in male than in female children. Therefore, no significant difference (*P* = 0.889) in the ratio of the CC area to the forebrain volume was identified between sexes in children.

Although the CC was slightly thicker in male than in female children in the genu (9.2 ± 1.6 vs. 8.5 ± 1.7 mm), body (5.3 ± 1.1 vs. 4.8 ± 0.9 mm), and splenium (13.8 ± 2.7 vs. 13.2 ± 2.1 mm), the differences were not statistically significant (*P* = 0.105, *P* = 0.114, and *P* = 0.326; respectively) for all regions.

#### Measurements in Younger Adults

There was no significant (*P* = 0.886) difference in the age between male and female younger adults. The mean ages for male and female younger adults were 32.5 ± 8.8 and 32.7 ± 8.8 years, respectively. No significant (*P* = 0.442) difference in the length of the CC was observed between male and female younger adults. The mean length of the CC was 68.8 ± 4.7 mm in males and 68.1 ± 3.5 mm in females. However, the mean length of the forebrain was significantly (*P* = 1.8 × 10^–8^) greater in younger male than in younger female adults (161.5 ± 6.7 vs. 153.3 ± 6.4 mm respectively). Therefore, the length ratio of the CC to the forebrain was significantly (*P* = 1.9 × 10^–4^) greater in younger female than in younger male adults.

No significant (*P* = 0.426) difference in the area of the CC was identified between younger male and younger female adults. The mean area of the CC was 592.1 ± 97.1 mm^2^ in younger adult males and 577.6 ± 74.0 mm^2^ in younger adult females. However, the forebrain volume was significantly (*P* = 3.6 × 10^–13^) greater in males than in females. This was reflected as a significantly (*P* = 7.9 × 10^–5^) greater ratio of the CC area to the forebrain volume in younger female than in younger male adults.

Lastly, no significant difference in thickness was identified between younger male and younger female adults in the genu (10.7 ± 1.4 vs. 11.0 ± 1.5 mm, *P* = 0.456), body (6.2 ± 0.8 vs. 6.1 ± 0.8 mm, *P* = 0.503), or splenium (17.0 ± 2.5 vs. 16.3 ± 2.2, *P* = 0.127) of the CC.

#### Measurements in Older Adults

There was no significant (*P* = 0.691) difference in the age between male and female older adults. The mean ages for male and female older adults were 63.3 ± 7.9 and 62.6 ± 7.0 years, respectively. The absolute length of the CC was significantly (*P* = 0.033) greater in older male than in older female adults. The mean lengths of the CC in older male and older female adults were 70.6 ± 3.4 mm and 68.5 ± 4.5 mm, respectively. In addition, the mean length of the forebrain was significantly (*P* = 7.0 × 10^–9^) greater in older male than in older female adults (161.0 ± 5.0 vs. 151.7 ± 6.6 mm, respectively). The length ratio of the CC to the forebrain length was significantly (*P* = 0.031) greater in older female than in older male adults.

No significant (*P* = 0.358) difference in the area of the CC was found between older male and older female adults. The mean area of the CC was 502.3 ± 79.8 mm^2^ in older adult males and 485.0 ± 76.1 mm^2^ in older adult females. However, the forebrain volume was significantly (*P* = 2.7 × 10^–11^) greater in males than in females. This was reflected as a significantly (*P* = 0.003) greater ratio of the CC area to forebrain volume in older female than in older male adults.

Finally, no significant difference in thickness was observed between older male and older female adults in the genu (9.3 ± 1.7 vs. 9.2 ± 1.8 mm, *P* = 0.702), body (5.4 ± 0.8 vs. 5.2 ± 0.8 mm, *P* = 0.376), or splenium (15.8 ± 2.6 vs. 15.5 ± 1.6 mm, *P* = 0.642) of the CC.

### Racial Comparisons

Both male and female racial comparisons showed similar trends in CC variation among different ethnicities (Tables 2, 3). No differences were found in the CC length (*P* = 0.403 for males and *P* = 0.642 for females) or area (*P* = 0.057 for males and *P* = 0.061 for females) between Polish and Arab populations. However, the CC was significantly longer (*P* = 1.6 × 10^–6^ for males and *P* = 1.9 × 10^–9^ for females) in Turkish people than in Arabs, despite the fact that the forebrain was significantly shorter (*P* = 2.6 × 10^–10^ for males and *P* = 3.1 × 10^–4^ for females) in the Turkish people. The shorter forebrain in the Turkish people resulted in significantly (*P* = 7.8 × 10^–17^ for males and *P* = 3.4 × 10^–17^ for females) greater length ratio of CC to forebrain in the Turkish people compared to Arabs.

**TABLE 2:** Racial comparison of the morphometric measurements of the corpus callosum in young adult males between Middle Eastern Arab and other ethnic backgrounds.

Ethnicity	Number of individuals	Forebrain length (mm)	CC length (mm)	Genu thickness (mm)	Body thickness (mm)	Splenium width (mm)	Total CC area (mm^2^)
Middle Eastern Arabs	40	161.5 ± 6.7	68.8 ± 4.7	10.7 ± 1.4	6.2 ± 0.8	17.0 ± 2.5	592.1 ± 97.1
Polish (Guz et al., 2019)	101	159.0 ± 7.5	69.4 ± 5.2	–	–	–	622.2 ± 86.5
Turkish (Karakaş et al., 2011)	23	152.5^↓↓^ ± 5.4	73.0^↑↑^ ± 5.3	13.2^↑↑^ ± 2.4	6.9^↑↑^ ± 2.1	11.9^↓↓^ ± 1.9	–

*Values are represented as the mean ± standard deviation. ^↑↑^P < 0.01 significantly greater than Arabs. ^↓↓^P < 0.01 significantly lesser than Arab measurements (One-sample t-test).*

**TABLE 3:** Racial comparisons of morphometric measurements of the corpus callosum in young adult females between Middle Eastern Arab and other ethnic backgrounds.

Ethnicity	Number of individuals	Forebrain length (mm)	CC length (mm)	Genu width (mm)	Body width (mm)	Splenium width (mm)	Total CC area (mm^2^)
Middle Eastern Arab	60	153.3 ± 6.4	68.1 ± 3.5	11.0 ± 1.5	6.1 ± 0.8	16.3 ± 2.2	577.6 ± 74.0
Polish (Guz et al., 2019)	101	151.5 ± 6.8	68.3 ± 5.0	–	–	–	595.8 ± 96.9
Turkish (Karakaş et al., 2011)	29	150.1^↓↓^ ± 5.0	71.3^↑↑^ ± 3.7	13.3^↑↑^ ± 2.1	7.6^↑↑^ ± 1.1	12.5^↓↓^ ± 1.4	–

*Values are represented as the mean ± standard deviation. ^↑↑^P < 0.01 significantly greater than Arabs. ^↓↓^P < 0.01 significantly lesser than Arab measurements (One-sample t-test).*

Furthermore, the CC was significantly thicker (*P* = 1.2 × 10^–5^ for males and *P* = 3.0 × 10^–20^ for females) in Turkish people than in Arabs, except in the splenium region. The CC was significantly (*P* = 7.1 × 10^–16^ for male and *P* = 3.5 × 10^–19^ for females) thicker in the region of the splenium in Middle Eastern Arabs than in the Turkish population (Tables 2, 3).

### Autism-Related Measurements

**FIGURE 3:**
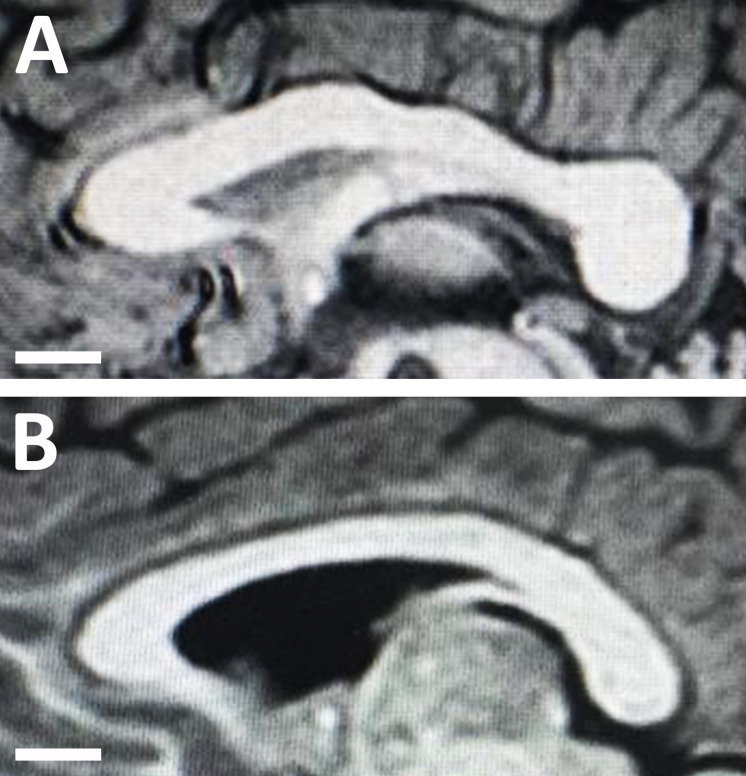
Midsagittal magnetic resonance images of the corpus callosum in **(A)** an 8-year-old neurotypical male child; and **(B)** an 8-year-old male child with autism spectrum disorder (ASD). Note the structural changes with autism disorder as the corpus callosum of the child with ASD appears much thinner compared to the callosum of the neurotypical child.

No significant (*P* = 0.141) difference in the length of the CC was found between neurotypical boys and boys with ASD. However, the area of CC was significantly (*P* = 0.002) smaller in boys with ASD compared to neurotypical boys (Table 4). Similarly, the CC was significantly thinner in the genu (*P* = 0.004), body (*P* = 0.001), and splenium (*P* = 2.2 × 10^–8^) of boys with ASD compared to neurotypical boys (Figure 3 and Table 4).

**TABLE 4:** Morphometric measurements of the corpus callosum (CC) between neurotypical boys and boys with autism spectrum disorder (ASD).

Measurement	Neurotypical (*n* = 28)	ASD (*n* = 21)
Age (y)	5.4 ± 2.1	7.6 ± 1.9
CC length (mm)	61.5 ± 4.0	63.9 ± 6.3
CC total area (mm^2^)	455.3 ± 83.8	381.9 ± 80.2**
**CC thickness (mm)**		
Genu	9.0 ± 1.5	7.8 ± 1.3**
Body	5.2 ± 1.1	4.3 ± 1.0**
Splenium	13.8 ± 2.7	9.2 ± 2.1**

Values are represented as the mean ± standard deviation (SD). ** P < 0.01 compared to neurotypical boys in the same row (independent t-tests).

## Discussion

This is the first morphometric study of the CC in the Middle Eastern Arab population, and, as such, it provides a reference for future comparisons with other populations, as well as for investigations of forensic and disease-related changes in the CC in Middle Eastern individuals. Several previous studies in the literature have described changes in the morphology of the CC in adults (Going and Dixson, 1990; Holloway et al., 1993; Pettey and Gee, 2002; Dubb et al., 2003; Suganthy et al., 2003) or children (Giedd et al., 1997; Tanaka-Arakawa et al., 2015), while few only have reported these changes across lifespan (Ardekani et al., 2013; Guz et al., 2019). This study uniquely analyzed data collected from Middle Eastern Arab individuals in three age groups, i.e., children, younger adults, and older adults. Our results show that the size of the CC increases significantly from childhood to adulthood and undergoes atrophic changes in thickness rather than length in older adults. Moreover, our finding of sex differences in the area of the CC in children suggests that sexual dimorphism is more likely to be determined by genetic factors than by hormonal or environmental factors. Prominent racial differences were also revealed by the morphometric measurements of the CC. In addition, this study demonstrated that children with autism have distinct changes in CC measurements. The morphometric analyses showed significant reductions in the area and thickness of CC in children with ASD compared to their neurotypical peers.

One source of confusion in morphometric analyses of the CC that may lead to inconsistent results and interpretations is the process of normalization (standardization) of CC measurements to brain size (Bermudez and Zatorre, 2001). Different normalization variables have been suggested as indices of brain size, particularly in studies of sexual dimorphism, such as the forebrain volume, brain weight, cranial capacity, and midsagittal intracranial area (Holloway et al., 1993; Bermudez and Zatorre, 2001; Sullivan et al., 2001). To avoid any such confusion in this study, we considered both the absolute and standardized values for the CC. The standardization method used in this study was the CC value relative to the forebrain volume. The forebrain volume was selected as our standardization variable because of the presence of significant linear and quadratic relationships between absolute CC measurements and forebrain volume, indicating a relatively homogenous enlargement of the CC with increased brain size (Jäncke et al., 1997).

**FIGURE 4:**
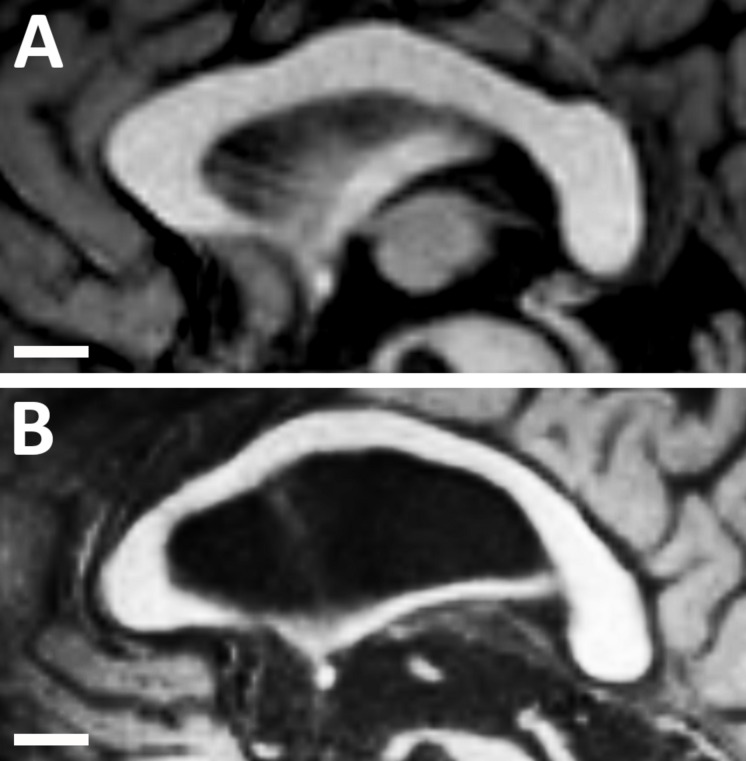
Midsagittal magnetic resonance images of the corpus callosum and lateral ventricle in **(A)** a healthy younger adult (aged 30 years); and **(B)** a healthy older adult (aged 68 years). Note the structural changes with age as the lateral ventricle becomes much larger and the corpus callosum becomes much thinner. However, the length of the corpus callosum remains approximately constant.

The anteroposterior lengths of the CC and forebrain were significantly shorter in children than in younger and older adults. This finding was expected given that growth and maturation of the CC and forebrain continue until one’s mid-twenties (Pujol et al., 1993). However, no difference in the length of the CC was found between younger and older adults. This finding contrasted with those of previous studies investigating other populations. For example, Suganthy et al. (2003) reported an increase in the length of the CC with age in an Indian population from the Vellore region, while other studies reported a decrease in CC length with age in the North American (US) population (Byne et al., 1988; Hopper et al., 1994). However, the relative length of the CC to the forebrain length was significantly larger in older adults than in younger adults in our study. This increase in the relative length with age may be explained by the expansion of the lateral ventricles which is associated with generalized brain atrophy in older adults. This expansion of the lateral ventricles could induce structural changes in the CC that may lead to a reduction in its thickness while maintaining its length, thereby preventing length reductions from occurring with age (Figure 4; Sullivan et al., 2002).

In our study, the area and thickness of the CC increased significantly from childhood to adulthood. Comparable results were reported in a previous study that investigated 114 Japanese subjects, including infants, children, adolescents, and adults (Tanaka-Arakawa et al., 2015). Barkovich and Kjos (1988) noted that the CC of newborns appears thin and flat and that the genu undergoes a large increase in thickness at 2 months of age and in the splenium between 4 and 6 months of age. It has been reported that the CC continues to develop together with the cortical brain areas after birth, specifically during the “growth spurt” period of the human cerebral cortex at ∼2 years of age (Matsuzawa et al., 2001; Tanaka-Arakawa et al., 2015). Furthermore, measurements of the total area of the CC in the younger adults in this study were comparable with those of previous MR imaging studies that included different populations (Clarke et al., 1989; Raine et al., 1990; Lacerda et al., 2005). However, a reduction in the area of the CC in older adults was observed when compared with younger adults. This reduction was expected given the brain atrophic changes that occur in older adults. As the brain ages, its sulci and ventricles become larger and the size of the CC decreases owing to the loss of cortical neurons (Going and Dixson, 1990). In addition, there is an age-related reduction in myelination, which would be expected to affect this heavily myelinated structure (Driesen and Raz, 1995).

In children, we found that the area of the CC was significantly larger in males than in females. To our knowledge, this is the first study to report a sex-related difference in CC size in children. This finding may support a previous suggestion that genetic factors play a more important role than environmental or hormonal factors in determining the size of the CC (Woldehawariat et al., 2014). Moreover, the forebrain volume was significantly larger in male than in female children, as is widely known to be the case (Giedd et al., 1997, 1999). The forebrain volume in this study was about 15% greater in males than in females. The proportional enlargement in both the CC and forebrain in male children resulted in no sex difference in the ratio of the CC area to forebrain volume.

No significant differences in the areas of the CC were identified between younger or older male and female adults. However, the forebrain volume was significantly larger in male subjects in both groups. The smaller forebrain volume in female subjects resulted in a significantly greater ratio of CC area to forebrain volume in younger and older female adults. Comparable findings have been reported in different populations (Jäncke et al., 1997; Tanaka-Arakawa et al., 2015).

With regard to racial comparisons, this study supports the concept that CC dimensions may vary across different ethnic and racial populations. This is comparable to what was reported by Nouri Hosseini et al. (2018), who highlighted that ethnicity may influence CC dimensions. However, it is very difficult to draw a solid conclusion regarding the racial variations in CC from our comparison study due to the presence of several limitations. First, the limited number of compared studies as we were able to retrieve only two studies that accomplished similar methodological criteria. Second, the lack of statistical power of the comparisons due to the small number of studied individuals from each population. Third, the unavailability of the raw data from the published studies. The presence of the raw data allows the performance of more comprehensive and more powerful statistical tests which can lead to more rigorous findings and conclusions. Finally, the inability to compare the standardized values of CC size according to the brain volume, as the observed variations might be related to differences in the brain volume rather than to differences in the CC itself.

Future studies are needed to determine the CC measurements across different ethnicities. This will have a greater clinical value, both in stereotaxic surgeries and for identifying abnormalities in these ethnicities. Therefore, we believe it is very important to have an international archive that provides reference data values, not only for CC, but for all brain measurements across different racial groups worldwide.

The presence of a correlation between the size of the CC and autism, and whether this correlation, if present, is causative or associative, remains unknown. Several studies reported no differences in CC size in patients with ASD compared to their neurotypical counterparts (Rice et al., 2005; Kucharsky Hiess et al., 2015; Lefebvre et al., 2015). In contrast, a growing body of literature supports the reduction in CC size in autism as a neuroanatomical sign of poor connectivity and reduced interhemispheric communication, leading to the impaired social and cognitive skills seen in autism (Frazier and Hardan, 2009; Keary et al., 2009; Casanova et al., 2011; Frazier et al., 2012; Prigge et al., 2013). In fact, more recent studies have emphasized that partial agenesis and hypoplasia of the CC could be a causative factor for autism (Wegiel et al., 2017; Patra et al., 2019). Regarding animal experiments on autism, the BTBR and BALB mice are the most widely accepted models since they provide the same core behavioral characteristics as autism (Varghese et al., 2017). Interestingly, the most prominent neuroanatomical feature in these animals is either the absence of CC, or the presence of a CC which is significantly reduced in size (Wahlsten et al., 2003; Fairless et al., 2008).

The exact nature of reduction in the CC size in patients with ASD remains poorly understood. However, it could be attributed to a smaller fiber size, decreased number of white fibers, and/or reduced myelination. A post-mortem study on human brains revealed a decrease in the number of large axons combined with an excessive increase in the number of thin axons within the white matter of patients with ASD (Zikopoulos and Barbas, 2010). In a recent study, Dimond et al. (2019) found a reduced fiber density in the corpus callosum of patients with ASD, suggesting that this reduced density may potentially reflect a decrease in the number of axons. In addition, they found the reduced fiber density in the region of the splenium to be associated with greater social impairment. With regard to myelination, there is a mounting evidence from the literature suggesting that autism is associated with hypomyelination and oligodendrocyte dysfunction (Wei et al., 2016; Kamata et al., 2017; Shen et al., 2018; Graciarena et al., 2019). A genomic-wide transcriptional study demonstrated defects in myelin stability and functionality genes in autism (Richetto et al., 2017). In addition, hypomyelination has been detected in different animal models of autism, including BTBR mice (Wei et al., 2016), mice exposed to valproic acid prenatally (Graciarena et al., 2019), and rats who had been exposed to both fetal inflammation and postnatal hypoxia (van Tilborg et al., 2018). Nevertheless, further studies are required to determine the exact nature of the reduction of CC size in patients with ASD and whether this reduction has a causative or associative relation to autism.

## Conclusion

This study shows that CC measurements vary according to age and sex in the Middle Eastern Arab population. With regard to age, the length of the CC was significantly smaller in children compared to both younger and older adults. However, no difference in CC length was observed between younger and older adults, and this finding may be attributed to the ventricular expansion which pressures the CC from inside, allowing it to maintain its length in older adults. In addition, the CC increased significantly in area and thickness from childhood to adulthood. However, significant reductions in these parameters were observed in older adults compared with younger adults, most likely owing to the physiological atrophic changes that occur during aging.

With regard to sexual dimorphism, the sizes of the CC and forebrain were generally larger in male than in female children. This indicates that heritable genetic factors are likely involved in determining the sizes of the CC and forebrain. No sex-related differences in CC area or thickness were observed in younger or older adults. However, the ratio of the CC area to the forebrain volume was generally greater in younger and older female adults due to their smaller forebrains.

Furthermore, this study supports the presence of racial variations in CC measurements. It also reveals that children with autism usually have a smaller CC compared to neurotypical children of the same ethnicity. Further investigations are warranted to determine the exact nature of the reduction in CC size within children with ASD.

## Data Availability Statement

The datasets generated for this study are available on request to the corresponding author.

## Ethics Statement

The studies involving human participants were reviewed and approved by the Institutional Review Board (IRB) Committee at Jordan University of Science and Technology. Written informed consent for participation was not required for this study in accordance with the national legislation and the institutional requirements.

## Author Contributions

MZA and MMA contributed to conception and design of the study. HA and SA collected the data and organized the database. MZA performed the statistical analysis. AM revised the study critically for important intellectual content. MZA, MMA, and HA wrote the sections of the manuscript. All authors contributed to manuscript revision, read and approved the submitted version.

## Conflict of Interest

The authors declare that the research was conducted in the absence of any commercial or financial relationships that could be construed as a potential conflict of interest.
